# The Pungent and Hot Chinese Herbs Cause Heat Syndrome in Rats by Affecting the Regulatory T Cells

**DOI:** 10.1155/2019/9824906

**Published:** 2019-07-09

**Authors:** De-Hong Wu, Li Xu, Guan-Qun Xie, Yong-Sheng Fan, Jia Zhou

**Affiliations:** ^1^The Second Affiliated Hospital of Zhejiang Chinese Medical University, 318 Chaowang Road, Hangzhou 310005, Zhejiang, China; ^2^College of Basic Medical Sciences, Zhejiang Chinese Medical University, 548 Binwen Road, Hangzhou 310053, Zhejiang, China

## Abstract

Heat syndrome is a folk saying in China, which is used to describe people with symptoms such as aphtha, oral ulcer, glossitis, swelling and aching of gingiva, and dry eye.* Aconitum carmichaelii *Debx. (A),* Zingiber officinale *Rosc. (Z), and* Cinnamomum cassia* Presl (C) are the representatives of pungent and hot Chinese herbs which may cause heat syndrome. In order to explore the mechanism of pungent herbs-induced heat syndrome, rats were treated with AZC extracts at different concentrations and at different time periods. A series of cytokines were determined using the cytokine antibody array; some immunosuppressive cytokines, including TGF-*β*, IL-10, and IL-35, significantly increased in AZC group as compared with control group. Higher mRNA expressions of Foxp3, TGF-*β*, IL-10, and IL-35 were found in the spleen and thymus of rats after treatment for 18 days based on RT-PCR. Flow cytometry result revealed that the percentage of CD4^+^CD25^+^ Treg cells and Foxp3^+^CD4^+^CD25^+^ Treg cells in spleen lymphocytes showed an increasing trend from the 3^rd^ day to the 18^th^ day after treatment with middle dose of AZC extracts. It is speculated that extracts of AZC herbs may affect the development of heat syndrome by influencing Treg cells and immunosuppressive cytokines.

## 1. Introduction

Traditional Chinese Medicine (TCM) has a long history in China dating back to several thousands of years ago and has formed its unique cognitive view of disease [1, 2]. Heat syndrome is a folk saying in China, which is used to describe people with symptoms such as aphtha, oral ulcer, glossitis, swelling and aching of gingiva, and dry eye. Heat syndrome is a common sub-health condition, but only a few researches have investigated the mechanism and the treatment of heat syndrome [3, 4].

Eating pungent and hot herbs/foods, fatigue, emotional upset, physical deterioration, and so on are the main causes of heat syndrome. Dried lateral roots of* Aconitum carmichaelii *Debx. (A), dried roots of* Zingiber officinale *Rosc. (Z), and dried bark of* Cinnamomum cassia* Presl (C) are the representatives of pungent and hot Chinese herbs in TCM, often used to treat the cold syndrome. Some healthy persons may develop heat syndrome after taking these herbs, because AZC herbs can support yang and generate excessive heat in TCM theory. Therefore, these herbs are always employed to establish animal models with heat syndrome [5].

In order to explore the mechanism of pungent herbs-induced heat syndrome, rats were treated with AZC herbs, and antibody array was employed to screen the significantly changed cytokines and make an assessment of immune function. Furthermore, inflammation-related cells and cytokines were investigated by RT-PCR and flow cytometry.

## 2. Materials and Methods

### 2.1. Reagents and Plant Materials

L Series 90: RayBio® Label-based Rat Antibody Array 1 (NO. AAR-BLM-1-2), RPMI-1640 culture medium (No. 11875093), and Trizol reagent (No. 15596-026) were purchased from Invitrogen (Carlsbad, CA). The PrimeScript RT-PCR kit (DRR014S), TaKaRa Taq™ (DR001A), and 100 bp DNA Ladder (3422A) were purchased from TaKaRa company (Dalian, China). Rat peripheral blood lymphocyte separation medium kit (No. LTS1083) was purchased from Haoyang Biological Manufacture (Tianjin, China). Anti-Mouse/Rat Foxp3 APC (No. 17-5773-82), PE Mouse anti-rat CD4 (No. 551397), and FITC Mouse anti-rat CD25 (No. 5561783) were purchased from Becton, Dickinson and Company (USA).

The raw herbal medicines for AZC preparation were purchased from the Chinese Herbal Medicine Co., Ltd. of Zhejiang Chinese Medical University (Hangzhou, China).

### 2.2. Preparation of Herbs Extract

The AZC formula used in the experiment was composed of dried lateral roots of* Aconitum carmichaelii *Debx., dried roots of* Zingiber officinale *Rosc., and dried bark of* Cinnamomum cassia* Presl. The three kinds of herbs were crushed and mixed together at a ratio of 1:1:1. After soaking in ultrapure water (w/v, 1/10) for 1 h, the mixed herbs were boiled for 2 h for extraction. The residue was extracted again for another 2 h. The filtrates were collected, combined, and concentrated to three concentrations (0.5 g, 1.0 g, 1.5 g crude drug/mL).

### 2.3. Animals and Treatment

The experimental protocol was approved by the Animal Welfare Committee of Zhejiang Chinese Medical University, Hangzhou, China (SYXK2008-0115). The animal housing facility is a barrier housing facility, and it is maintained in accordance with the national standards (Laboratory Animal-Requirements of Environment and Housing Facilities, GB 14925-2001). Sprague-Dawley (SD) rats (weighing 150±20 g, 96 males) were obtained from the Laboratory Animal Research Center of Zhejiang Chinese Medical University.

12 rats were divided into AZC group and control group with 6 in each group. Two groups were given, by gavage twice a day for 14 days, AZC extract (1.0 g crude drug/mL) or ultrapure water, 5 mL/kg per day, respectively. One hour after the last administration, the blood of rats in the AZC group and control group was sterilely collected through the celiac vein. After settling for 3-4 h at room temperature, the rat serum was separated by centrifugation at 3000 r/min at 4°C for 15 min and then stored at -80°C for serum cytokine profiling assay.

In order to investigate the time-dependent effect of AZC herbs, 60 rats were randomly divided into AZC group and control group and orally administered moderate-dose AZC extract (1.0 g crude drug/mL) or ultrapure water over different times (3, 9, 18, 27, and 36 days). One hour after the last administration, the rats were paunched to obtain spleens and thymus glands under 1% chloral hydrate anesthesia. Thymus and spleen are important immune organs, and their organ indexes are rough indicators of the body immunity. The spleen and thymus indexes of each rat were measured by dividing the weight of the spleen/thymus gland (mg) by the body weight (g) and multiplying by 10.

To investigate the dose-dependent effect of AZC herbs, rats were administered different concentrations of AZC extract (0.5 g, 1.0 g, 1.5 g crude drug/mL) or ultrapure water for 18 days via gastrogavage, respectively (6 in each group). One hour after the last administration, the rats were paunched to obtain spleens and thymus glands under 1% chloral hydrate anesthesia, and then the spleen and thymus indexes of rats were measured.

### 2.4. Serum Cytokines Screening by Antibody Array

A series of cytokines were determined using the rat cytokine antibody array kit according to the standard procedure of the RayBiotech kit. In brief, the array membranes were blocked and afterward incubated with serum samples overnight. Then, the membranes were incubated with biotin-conjugated antibodies at room temperature. After washing, incubation with Cy3-conjugated-streptavidin was conducted. The signals were detected by fluorescence and the relative levels of the cytokines were quantified by densitometry.

### 2.5. Foxp3, TGF-*β*, IL-10, and IL-35 mRNA Levels in Rats Spleen and Thymus Determined by RT-PCR

Total RNA was extracted from the cell layer of each group with Trizol reagent. The quantity and purity of RNA were determined by measuring the absorbance at 260 and 280 nm using a spectrophotometer (Unico Co., USA). The total RNA was reverse transcribed into complementary DNA (cDNA) with a cDNA synthesis kit and amplified in a PCR machine (Eppendorf, Germany). The specific primers for the target genes and GAPDH (synthesized by Shanghai Shenggong Co.) were used as described in Table 1.

A two-step PCR procedure was recommended as follows: predenaturation for 10 s at 94°C, 1 cycle; 94°C for 30 s, 54°C for 30 s, and 72°C, 10 min, 30 cycles. The final products were identified by electrophoresis on a 1% agarose gel and analyzed with an automatic image analyzer. The mRNA level of GAPDH was used as an internal control, and gene-specific mRNA expression was normalized against *β*-actin expression. Data is presented as mean ± SD of triplicate measurements

### 2.6. Percentage of CD4^+^CD25^+^ and Foxp3^+^CD4^+^CD25^+^ Treg Cells in Rats Spleen Lymphocytes Determined by Flow Cytometry

The spleens were cut into pieces, washed with RPMI-1640 culture medium, and centrifuged for 5 min at 1500r/min. After filtering with 100-mesh nylon mesh, the supernatant was discarded, and the spleen cells of each group were suspended with RPMI-1640 culture medium, and then rat spleen lymphocytes were isolated and collected by using a rat peripheral blood lymphocyte separation medium kit.

Isolated rat spleen lymphocytes were washed twice with PBS containing 1% fetal calf serum (FCS) and then resuspended in it at a concentration of 1 x 10^6^ cells/ml. One hundred microliters of the solution of each group was transferred into a 5 mL culture tube; then 5 *μ*l of PE Mouse anti-rat CD4 mAb and FITC Mouse anti-rat CD25 mAb was added. The cells were gently vortexed and incubated for 30 min at room temperature in the dark. Next, 400 *μ*l of PBS containing 1% FCS was added to each tube. The prepared samples were analyzed by flow cytometry (Beckman Cytomics FC 500) within 1 hour.

### 2.7. Statistical Analysis

All values were expressed as the mean ± standard deviation. One-way ANOVA was used for comparison between multiple groups (>2). Comparison between 2 groups was done with* t*-test. Differences were considered statistically significant if the* p* value was less than 0.05. Statistical analysis was carried out by SPSS 16.0.

## 3. Results

### 3.1. Effect of AZC on the Body Weight of Rats

**Figure 1:**
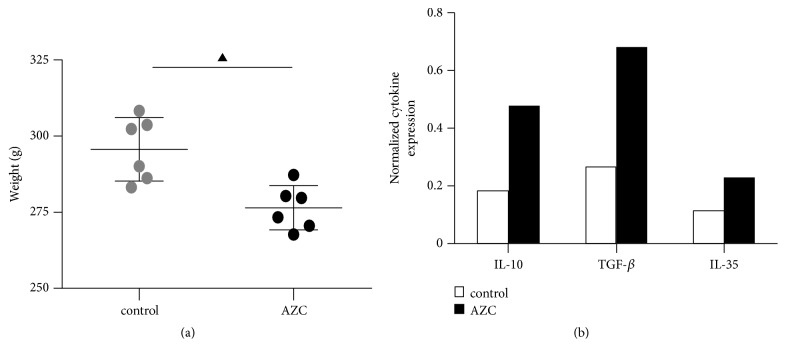
(a) Effect of AZC on the body weight of rats, (b) abnormally expressed serum cytokines in AZC rats measured by antibody array. ▲*p*<0.05 compared to the control group.

During the experiment, the general state of rats was observed and compared. As compared to the control group, the hair of AZC group seemed tanglesome and lustrous, the stool was forming but a little dry, and the tongue and gingiva color was light red. The body weight of the rats decreased significantly after treatment with AZC extract for 14 days (*p*<0.05, Figure 1(a)).

### 3.2. Effect of AZC on the Serum Cytokine Profiles of Rats

The serum cytokine profiles were measured by antibody array, which indicated that some cytokines, including IL-10, TGF-*β*, and IL-35, were abnormally expressed in AZC groups with more than twofold increase as compared with control group (Figure 1(b)). IL-10, TGF-*β*, and IL-35 are important immunosuppressive factors which can be secreted by Treg cells. Therefore, we paid particular attention to Treg cells in further study.

### 3.3. Time-Dependent Effect of AZC on the Rats Body Weight, Spleen, and Thymus Indexes

**Figure 2:**
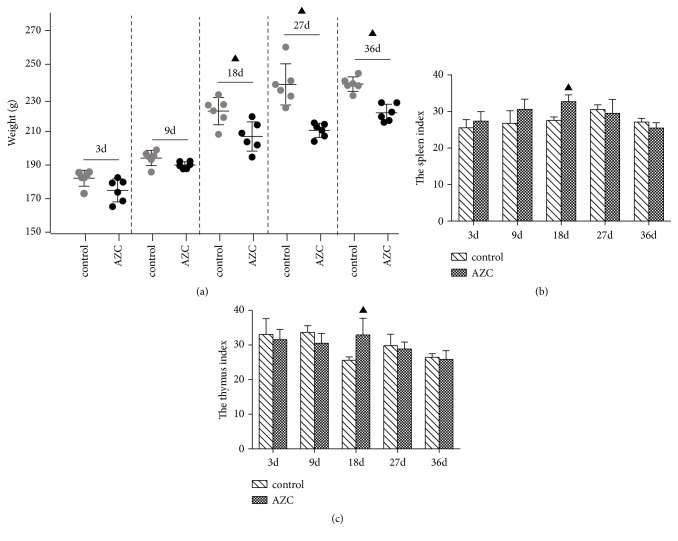
Time-dependent effect of AZC on the body weight of rats (a), spleen index (b), and thymus index (c). ▲*p*<0.05 compared to the control group.

The body weight of rats decreased significantly in AZC group on the 18th day, 27th day, and 36th day compared to the corresponding control group (*p*<0.05, Figure 2(a)). The spleen and thymus indexes of the rats increased significantly in AZC group on the 18th day (*p*<0.05), but the indexes did not change significantly on the 3rd day, 9th day, 27th day, and 36th day compared to the corresponding control groups (Figures 2(b) and 2(c)).

### 3.4. Time-Dependent Effect of AZC on mRNA Expression of Foxp3, TGF-*β*, IL-10, and IL-35 in Rats Spleen and Thymus

**Figure 3:**
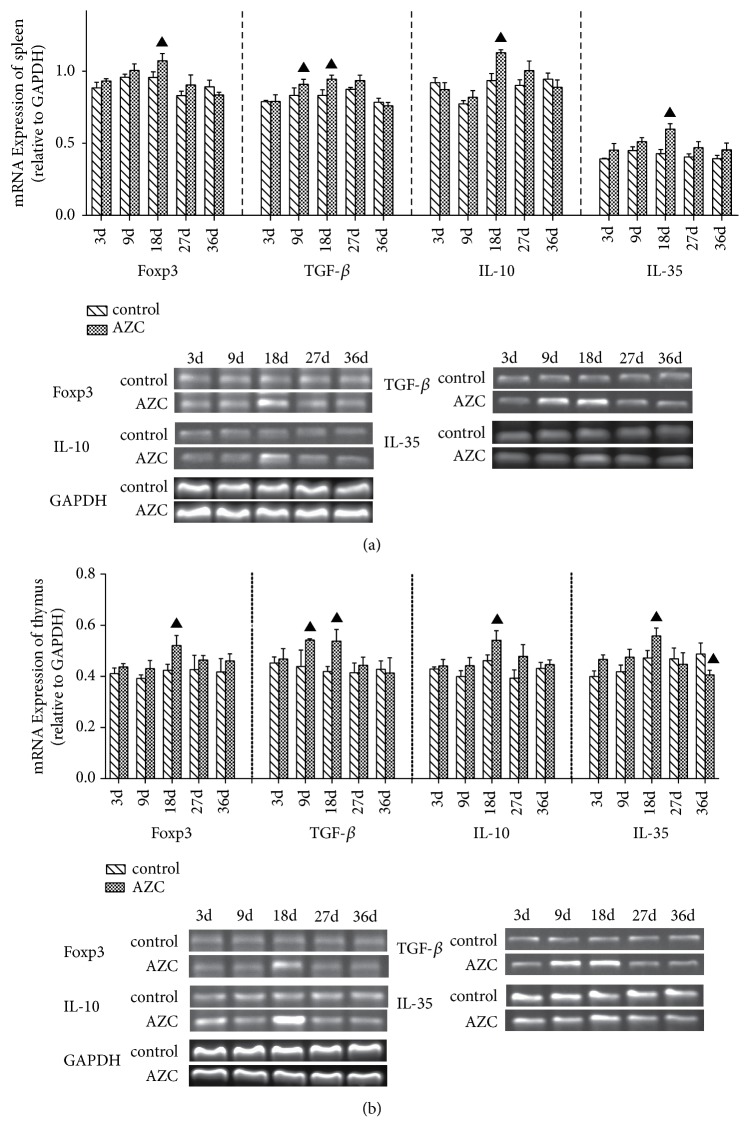
Time-dependent effect of AZC on mRNA expression of Foxp3, TGF-*β*, IL-10, and IL-35 in rat spleen (a) and thymus (b). The mRNA expression was determined by RT-PCR, the mRNA level of GPDH was used as an internal control, and gene-specific mRNA expression was normalized against GPDH expression. Results are shown as means ± SD. ▲*p*<0.05 compared to the control group.

Treg play a fundamental role in controlling the immune responses. Foxp3 is one of the key transcription factors regulating the development and differentiation of Treg cells. The effects of AZC herbs on the mRNA expression of Foxp3, TGF-*β*, IL-10, and IL-35 in the spleen and thymus were determined by RT-PCR (Figure 3). The mRNA expression of TGF-*β* in rats spleen and thymus increased significantly after treatment with AZC for 9 days (*p*<0.05), and mRNA expressions of Foxp3, IL-10, and IL-35 increased significantly after treatment with AZC for 18 days (*p*<0.05). However, Foxp3, TGF-*β*, and IL-10 did not increase significantly on the 27th day and 36th day in spleen and thymus, and the IL-35 decreased significantly on the 36th day in rats spleen (*p*<0.05).

### 3.5. Time-Dependent Effect of AZC on the Percentage of CD4^+^CD25^+^ and Foxp3^+^CD4^+^CD25^+^ Treg Cells in Rats Spleen Lymphocytes

**Figure 4:**
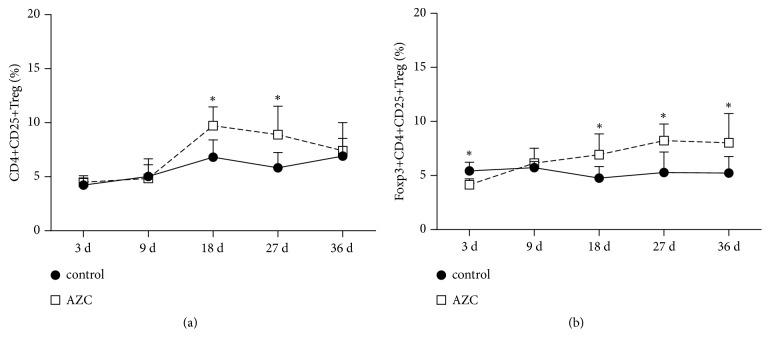
Time-dependent effect of AZC on the percentage of CD4^+^CD25^+^ Treg cells (a) and Foxp3^+^CD4^+^CD25^+^ Treg cells (b) in rat spleen lymphocytes. Percentage of Treg cells in rat spleen lymphocytes was determined by flow cytometry. Results are shown as means ± SD. *∗p*<0.05 means compared to the control group.

The percentage of CD4^+^CD25^+^ Treg cells remarkably increased on the 18th day and 27th day after treatment with AZC (*p*<0.05, Figure 4(a)). Similarly, the percentage of Foxp3^**+**^CD4^+^CD25^+^ Treg cells increased significantly after treatment with AZC for 18 days (*p*<0.05, Figure 4(b)).

### 3.6. Dose-Dependent Effect of AZC on the Rats Body Weight, Spleen, and Thymus Indexes

**Figure 5:**
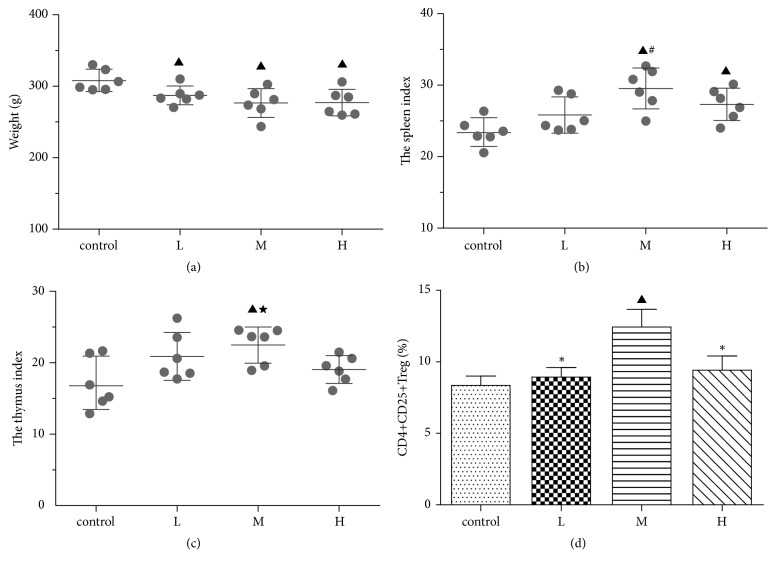
Dose-dependent effect of AZC on the body weight (a), spleen index (b), thymus indexes (c), and percentage of CD4^+^CD25^+^ Treg cells in rat spleen lymphocytes (d). Percentage of CD4^+^CD25^+^ Treg cells in rat spleen lymphocytes was determined by flow cytometry. L, M, and H represent the low-, moderate-, and high-dose AZC group, respectively. ▲*p*<0.05 compared to the control group, ★* p*<0.05 compared to the H group, #* p*<0.05 compared to the L group, *∗ p*<0.05 compared to the M group.

Rats were administered different doses (low, moderate, and high) of AZC extract corresponding to L group, M group, and H group, respectively. The changes of body weight (Figure 5(a)), spleen index (Figure 5(b)), and thymus index (Figure 5(c)) in three AZC groups were measured. The body weight of the AZC-treated rats decreased markedly on the 18th day compared to the control group (*p*<0.05), but there were no significant differences between the three dosage groups. The spleen indexes of the rats increased significantly in the M and H groups on the 18th day compared to the control group (*p*<0.05), but did not change significantly in the L group. The spleen indexes of the rats were much higher in the M group than in the L group (*p*<0.05). The thymus indexes of the rats increased significantly in the M group on the 18th day compared to the control group (*p*<0.05), but did not change significantly in the L and H groups.

### 3.7. Dose-Dependent Effect of AZC on the Percentage of CD4^+^CD25^+^ Treg Cells in Rats Spleen Lymphocytes

The percentage of CD4^+^CD25^+^ Treg cells in rat spleen lymphocytes increased significantly in the M group as compared to the control group (*p*<0.05), while it was not notably changed in the L and H groups (Figure 5(d)). And the percentage was much higher in the M group than in the L and H groups (*p*<0.05, Figure 5(d)).

## 4. Discussion

Main symptoms of heat syndrome are manifested on the head and face, including aphtha, oral ulcer, glossitis, and swollen gums. A few researchers have explored the major manifestations of TCM heat syndrome, such as oral ulcer [6], but the biological essence of heat syndrome is not completely understood yet. In the study, AZC herbs were used to set up heat syndrome rat model [5, 7], and we observed manifestation of heat syndrome. The hair of AZC-treated rat was tanglesome, a little yellow, and lacking in lustrousness; the stool was forming but dry; and the tongue and gingiva color was light red as compared to the control group. After comparing the serum cytokine profiles, it was revealed that the levels of TGF-*β*, IL-10, and IL-35 were abnormally increased in AZC group by more than 2-fold. These cytokines play an extremely important role in the differentiation, development, and proliferation of Treg cells and maintaining immunosuppression and tolerance of Treg cells [8–10].

Treg cells is a subset of T cells which can inhibit immune responses [11], and it has been reported that CD4^+^CD25^+^ Treg cells can inhibit the proliferation and activation of T effector cells to reduce the response to foreign or autoantigen, keeping the immune tolerance of body [12, 13]. According to the different sources, Treg cells can be divided into natural Treg cells (nTreg) and induced Treg cells (iTreg) [14]. nTreg cells mainly come from direct differentiation of T cells in the thymus and the lower expression of CD45RB in this subset of T cells. iTreg cells, which are generally induced by antigen or other factors (such as TGF-*β* stimulation), mainly come from the differentiation of CD4^+^ T cells, and their phenotype is coincident with nTreg cells [13, 15].

Two major functions of CD4^+^D25^+^ Treg cells are immunosuppression and immune incompetence. Immunosuppression of CD4^+^CD25^+^ Treg cells mainly manifests itself in suppressing T cells proliferation and differentiation, inhibiting the antigen presented cells (APC) to present antigen and mediating target cells apoptosis directly; immunosuppressive effects can be mediated by the intercellular contacts or secretion of related cytokines( IL-10, TGF-B, etc.) without the restrictions of MHC [15, 16]. CD4^+^CD25^+^ Treg cells showed no response to the stimulation of high level of IL-2, anti CD3 monoclonal antibody, or the combination of anti CD3 and anti CD28 monoclonal antibody, and it was considered as an important manifestation of immune incompetence of CD4^+^CD25^+^ Treg cells.

CD4^+^CD25^+^ Tregs have been confirmed to play a critical role in maintaining immune balance and immunologic homeostasis [17], but the molecular basis of their development and function remains incompletely understood. The Treg-specific gene, forkhead box protein P3 (Foxp3), encodes a transcription factor, which is exclusively expressed in CD4^+^CD25^+^ Treg cells [18]. There is compelling evidence that Foxp3 serves as a lineage specification factor for Treg cells, and Foxp3 is described as a key control gene for both the development and the function of CD4^+^CD25^+^ Treg cells [19–21]. Loss-of-function studies confirmed that although CD4^+^CD25^+^ Treg cells were present in the periphery of Foxp3^−^ deficient mice, they were neither anergic nor suppressive in vitro [19, 21]. In addition, retroviral gene transfer of Foxp3 converted human CD4^+^CD25^−^T cells toward a regulatory phenotype in vitro, and similar result was found in animal experiment [22].

In the study, it was found that the rise of mRNA expression of Foxp3 was accompanied by the increase of spleen and thymus indexes in rats after treatment with moderate-dose AZC for 18 days. And flow cytometry revealed that the percentage of CD4^+^CD25^+^ Treg cells and Foxp3^+^CD4^+^CD25^+^ Treg cells in rat spleen lymphocytes showed a trend of gradually increase from 3 days to 18 days, especially 18 days, after treatment with moderate-dose AZC.

TGF-*β* could induce the gene expression of Foxp3 in mouse CD4^+^CD25^–^T cells, converting them into Foxp3^+^ Treg cells [23]. Additionally, this has been independently confirmed by many studies and expanded to be applied to human T cells, where it has been indicated that TGF-*β* can mediate the generation of Treg cells [24, 25]. Nowadays, many experimental approaches and methods have been developed in an effort to generate Treg cells. TGF-*β* is regarded as essential for the generation of Treg cells from CD4^+^ T cells [26]. Our experiment showed that AZC herbs effectively increased the levels of TGF-*β* in the spleens and thymuses of rats after treatment for 9 days and 18 days, especially 18 days.

Treg cells are believed to play an important role in regulating inflammatory responses by mediating immunosuppressive cytokines that facilitate immune suppression [13, 27–30], and IL-10 has been implicated in Treg cells-mediated suppression. However, the precise role of IL-10 remains controversial [31–33]. Recent studies indicated that IL-10 may contribute to Treg cells-mediated suppression by inhibiting the expansion and effector functions of self-reactive T cells, and it could exert an effect on self-reactive T cells directly or, through DCs, indirectly [34, 35]. In the study, we found that the treatment of AZC effectively increased the levels of IL-10 in the spleens and thymuses of rats after 18 days, and the result was consistent with the trend of Foxp3 and TGF-*β*.

IL-35 is the newest member of the IL-12 family, which has been identified as a novel immunosuppressive/anti-inflammatory cytokine that is produced by Treg cells and contributes to the suppressive activity [10, 36, 37]. IL-35 is secreted by Treg cells, but not conventional T cells, and is required for maximal Treg function in vitro and in vivo [10]. Recent studies also found that ectopic expression of IL-35 by conventional T cells is sufficient to confer regulatory activity, but that recombinant IL-35 can suppress the proliferation of anti-CD3 stimulated conventional T cells [38]. Our study suggested that AZC herbs significantly increased the mRNA expression of IL-35 in the spleens and thymuses of rats after treatment for 18 days.

## 5. Conclusion

AZC herbs are the representatives of pungent and hot Chinese herbs which may cause heat syndrome in TCM theory. This study revealed that some immunosuppressive cytokines, such as TGF-*β*, IL-10, and IL-35, were significantly increased in the serum of AZC-treated rats. The mRNA expressions of Foxp3, TGF-*β*, IL-10, and IL-35 in the spleens and thymuses were significantly increased after the treatment with AZC herbs for 18 days. Also, the percentages of CD4^+^CD25^+^ Treg cells and Foxp3^+^CD4^+^CD25^+^ Treg cells in rat spleen lymphocytes were increased. It is possible that the changes of Treg cells and immunosuppressive cytokines may play an important role in the onset and development of heat syndrome induced by pungent and hot Chinese herbs.

## Figures and Tables

**Table 1:** Primers for the target genes and *β*-actin used in RT-PCR.

Target gene	Primer	Length of Target fragment (bp)
Foxp3	5′- TGCTGGCAAACGGAGTC -3′ (F)	401
	5′- GGGTGGCATAGGTGAAA -3′ (R)	
TGF-*β*	5′- GTAGCCACCAGCACCCA -3′ (F)	356
	5′- TCCACTCGCACAAAGCAC -3′ (R)	
IL-10	5′- CTACCATAGCCACAACGC -3′ (F)	325
	5′- GCAACCCAAGTAACCCTTA -3′ (R)	
IL-35	5′-ATGGCTAGGCTCTGTGCTTTC-3′ (F)	344
	5′-TGGGCATCCACCTTCTCC-3′ (R)	
GAPDH	5′- GCCTCACTTCCTACCCTCG -3′ (F)	514
	5′- TCAGGATACCTCGCTTGCT -3′ (R)	

## Data Availability

The data used to support the findings of this study are included within the article.

## References

[1] Tang J. L., Liu B. Y., Ma K. W. (2008). Traditional Chinese medicine. *The Lancet*.

[2] Stone R. (2008). Biochemistry. lifting the veil on traditional chinese medicine. *Science*.

[3] Liu C.-M., Chen J., Yang S. (2018). iTRAQ-based proteomic analysis to identify the molecular mechanism of Zhibai Dihuang Granule in the Yin-deficiency-heat syndrome rats. *Chinese Medicine*.

[4] Liu Y. M. (2005). Comparative study on T-lymphocyte subsets in the patients of excess-heat syndrome and deficiency-heat syndrome. *Journal of Traditional Chinese Medicine*.

[5] Li L., Dai X., Li B., Yang W., Wang X., Huang L. (2013). Effects of interior-warming herbs on the change of rats heatsyndrome. *Pharmacology and Clinics of Chinese Materia Medica*.

[6] Qin L., Li Y., Jiao Y. (2016). Changes in blood components in aphtha patients with excess heat. *Evidence-Based Complementary and Alternative Medicine*.

[7] Xu Z., Chen Q., Sun Q., Liu Y., Wang B., Chen Z. (2009). Investigation of essence of TCM heat syndrome. *Chinese Journal of Comparative Medicine*.

[8] Zhang H., Podojil J. R., Chang J., Luo X., Miller S. D. (2010). TGF-*β*–induced myelin peptide-specific regulatory T cells mediate antigen-specific suppression of induction of experimental autoimmune encephalomyelitis. *The Journal of Immunology*.

[9] Maloy K. J., Salaun L., Cahill R., Dougan G., Saunders N. J., Powrie F. (2003). CD4^+^CD25^+^ TR cells suppress innate immune pathology through cytokine-dependent mechanisms. *The Journal of Experimental Medicine*.

[10] Collison L. W., Workman C. J., Kuo T. T. (2007). The inhibitory cytokine IL-35 contributes to regulatory T-cell function. *Nature*.

[11] Sakaguchi S., Yamaguchi T., Nomura T., Ono M. (2008). Regulatory T cells and immune tolerance. *Cell*.

[12] Asano M., Toda M., Sakaguchi N., Sakaguchi S. (1996). Autoimmune disease as a consequence of developmental abnormality of a T cell subpopulation. *The Journal of Experimental Medicine*.

[13] Sakaguchi S., Sakaguchi N., Asano M., Itoh M., Toda M. (1995). Immunologic self-tolerance maintained by activated T cells expressing IL-2 receptor alpha-chains (CD25). breakdown of a single mechanism of self-tolerance causes various autoimmune diseases. *Journal of Immunology*.

[14] Cassis L., Aiello S., Noris M. (2005). Natural versus adaptive regulatory T cells. *Contributions to Nephrology*.

[15] Shevach E. M. (2002). CD4^+^CD25^+^ suppressor T cells: more questions than answers. *Nature Reviews Immunology*.

[16] Sakaguchi S. (2003). The origin of FOXP3-expressing CD4^+^ regulatory T cells: thymus or periphery. *The Journal of Clinical Investigation*.

[17] Curotto de Lafaille M. A., Lafaille J. J. (2002). CD4(+) regulatory T cells in autoimmunity and allergy. *Current Opinion in Immunology*.

[18] Hori S., Takahashi T., Sakaguchi S. (2003). Control of autoimmunity by naturally arising regulatory CD4+ T cells. *Advances in Immunology*.

[19] Fontenot J. D., Gavin M. A., Rudensky A. Y. (2003). Foxp3 programs the development and function of CD4+CD25+ regulatory T cells. *Nature Immunology*.

[20] Hori S., Nomura T., Sakaguchi S. (2003). Control of regulatory T cell development by the transcription factor Foxp3. *Science*.

[21] Khattri R., Cox T., Yasayko S., Ramsdell F. (2003). An essential role for Scurfin in CD4+CD25+ T regulatory cells. *Nature Immunology*.

[22] Yagi H., Nomura T., Nakamura K. (2004). Crucial role of *FOXP3* in the development and function of human CD25^+^CD4^+^ regulatory T cells. *International Immunology*.

[23] Chen W., Jin W., Hardegen N. (2003). Conversion of peripheral CD4+CD25- naive T cells to CD4+CD25+ regulatory T cells by TGF-beta induction of transcription factor Foxp3. *The Journal of Experimental Medicine*.

[24] Horwitz D. A., Zheng S. G., Wang J., Gray J. D. (2008). Critical role of IL-2 and TGF-*β* in generation, function and stabilization of Foxp3^+^CD4^+^ Treg. *European Journal of Immunology*.

[25] Davidson T. S., DiPaolo R. J., Andersson J., Shevach E. M. (2007). Cutting edge: IL-2 is essential for TGF-*β*-mediated induction of Foxp3^+^ T regulatory cells. *The Journal of Immunology*.

[26] Chen W., Konkel J. E. (2015). Development of thymic Foxp3(+) regulatory T cells: TGF-beta matters. *European Journal of Immunology*.

[27] Maloy K. J., Powrie F. (2001). Regulatory T cells in the control of immune pathology. *Nature Immunology*.

[28] Powrie F., Correa-Oliveira R., Mauze S., Coffman R. L. (1994). Regulatory interactions between CD45RBhigh and CD45RBlow CD4+ T cells are important for the balance between protective and pathogenic cell-mediated immunity. *The Journal of Experimental Medicine*.

[29] Sakaguchi S., Sakaguchi N., Shimizu J. (2001). Immunologic tolerance maintained by CD25^+^ CD4^+^ regulatory T cells: their common role in controlling autoimmunity, tumor immunity, and transplantation tolerance. *Immunological Reviews*.

[30] Shevach E. M. (2000). Suppressor T cells: rebirth, function and homeostasis. *Current Biology*.

[31] Green E. A., Gorelik L., McGregor C. M., Tran E. H., Flavell R. A. (2003). CD4+CD25+ T regulatory cells control anti-islet CD8+ T cells through TGF-beta-TGF-beta receptor interactions in type 1 diabetes. *Proceedings of the National Acadamy of Sciences of the United States of America*.

[32] Powrie F., Carlino J., Leach M. W., Mauze S., Coffman R. L. (1996). A critical role for transforming growth factor-beta but not interleukin 4 in the suppression of T helper type 1-mediated colitis by CD45RB(low) CD4+ T cells. *Journal of Experimental Medicine*.

[33] Suri-Payer E., Cantor H. (2001). Differential cytokine requirements for regulation of autoimmune gastritis and colitis by CD4+CD25+ T cells. *Journal of Autoimmunity*.

[34] Annacker O., Pimenta-Araujo R., Burlen-Defranoux O., Barbosa T. C., Cumano A., Bandeira A. (2001). CD25+CD4+ T cells regulate the expansion of peripheral CD4 T cells through the production of IL-10. *Journal of Immunology*.

[35] Huang X., Zhu J., Yang Y. (2005). Protection against autoimmunity in nonlymphopenic hosts by CD4+ CD25+ regulatory T cells is antigen-specific and requires IL-10 and TGF-beta. *The Journal of Immunology*.

[36] Cai Z., Wong C. K., Kam N. W. (2015). Aberrant expression of regulatory cytokine IL-35 in patients with systemic lupus erythematosus. *Lupus*.

[37] Collison L. W., Vignali D. A. A. (2008). Interleukin-35: odd one out or part of the family?. *Immunological Reviews*.

[38] Collison L. W., Pillai M. R., Chaturvedi V., Vignali D. A. A. (2009). Regulatory T cell suppression is potentiated by target T cells in a cell contact, IL-35- and IL-10-dependent manner. *The Journal of Immunology*.

